# Cellulose-Based Oleogels via One-Step Cross-Linking for Lubrication

**DOI:** 10.3390/molecules31142538

**Published:** 2026-07-21

**Authors:** Yuhao Fang, Gaobo Lou, Hongjiang Yu, Lina Liu, Yifan Chen

**Affiliations:** 1 College of Chemistry and Materials Engineering, Zhejiang A&F University, Hangzhou 311300, China; 18839969651@163.com (Y.F.); 13151320460@163.com (H.Y.); 2 Zhejiang Key Laboratory of Green and Low-Carbon Utilization Technology of Agriculture and Forestry Biomass, Hangzhou 311300, China

**Keywords:** cellulose, epoxidized soybean oil, oleogels, rheology, tribology

## Abstract

In this study, novel and stable cellulose-based oleogels with tunable rheological properties were successfully developed for lubrication applications via cross-linking reactions of epoxidized soybean oil (ESO), microcrystalline cellulose (MCC), and isocyanate. This cross-linking strategy not only overcomes the incompatibility issue arising from the polarity difference between MCC and ESO but also enables precise control over the oleogels’ rheological behavior by tailoring the cross-linking density. The resulting oleogels exhibit excellent thermal stability, with an initial decomposition temperature (*T*_5%_) of approximately 300 °C. Furthermore, oxidation resistance is significantly enhanced with increasing cross-linking density, resulting in a substantial increase in the oxidation induction time (OIT) from 5 to 79 min at 210 °C. Rheological characterization reveals that the oleogels exhibit typical shear-thinning and thixotropic behavior. The plateau modulus ($G_{N}^{0}$) exhibits a positive correlation with cross-linking density, accompanied by a simultaneous improvement in structural recovery ability. Tribological tests show that the friction coefficient increases with the cross-linking degree, while four-ball tests indicate that the extreme-pressure load-carrying capacity is governed mainly by the nature of the base oil in addition to the cross-linking density of the gel network. This work provides a promising strategy for the development of high-performance and customizable bio-based lubricating materials.

## 1. Introduction

Gels, as biphasic systems comprising a dispersed phase within a dispersion medium, are extensively used spanning daily life to industrial processes [1,2,3]. Oleogels—formed by structuring liquid oils into semi-solid matrices—have attracted considerable attention for their diverse applications, which can be broadly classified into two main categories. One is in healthcare, food, and cosmetics, where they serve as fat replacers, controlled-release carriers, and texture modifiers [4,5,6]. The other is in industrial lubrication, where their inherent lubricity makes them promising candidates functionally analogous to conventional greases [7,8]. However, commercial greases are predominantly petroleum-based, relying on non-biodegradable mineral oils and metal soap thickeners [9]. Their production and lifecycle raise pressing concerns regarding carbon emissions, feedstock sustainability, and ecological toxicity [10,11]. In contrast, bio-based materials derived from renewable resources offer a compelling alternative, boasting advantages such as low toxicity, environmental compatibility, and biodegradability. Consequently, the development of bio-based oleogels for lubrication has emerged as a vibrant research frontier [12,13].

Currently, bio-based oleogels are usually prepared using vegetable oils such as castor oil [14,15], soybean oil [16,17], and rapeseed oil [18]. For instance [19], Adhvaryu et al. prepared oleogels using soybean oil as the base oil and lithium soap as the thickener. Subsequently, the effects of lithium soap type and addition amount on the physicochemical properties of the oleogels were investigated. Rawat et al. [20] conducted a similar study, using castor oil as the base oil and lithium soap as the thickener to prepare bio-based oleogels. The tribological properties of the oleogels were investigated by incorporating MoS_2_ and graphene oxide nanosheets as additives. However, it is not sufficiently environmentally friendly to use metal soaps as thickeners for preparing oleogels, as this can cause heavy metal pollution to the environment. Thus, various bio-based materials, such as cellulose [21], lignin [22], and chitosan [23], have been reported to replace metal soaps for thickening vegetable oils in recent years. Gallego et al. [24] prepared bio-based oleogels using castor oil as the base oil and HMDI-modified methylcellulose as the thickener. The -NCO groups of HMDI react with the hydroxyl groups of methylcellulose to form carbamate bonds (-NH-CO-O-), thereby reducing the polarity of methylcellulose and enhancing its affinity with castor oil. Similarly, Wu et al. [25] prepared a lignin-castor oil-based oleogels using castor oil as the base oil and silane coupling agent-modified lignin as a thickener. The prepared oleogels exhibited excellent lubricating and antioxidant properties comparable to those of commercial greases. It follows that bio-based oleogels have the potential to replace commercial greases as promising lubricating materials.

Cellulose is a natural polysaccharide compound existing in plant biomass and also produced by certain bacteria, and it is the most abundant biopolymer material on earth, which offers advantages of low cost, biodegradability, and non-pollution to the environment [26,27,28]. It can be used as a thickener in daily chemical products [29], food [30], medicine [31], and other fields for viscosity adjustment. Therefore, among various bio-based materials, using cellulose as a thickener to prepare oleogels by thickening vegetable oils holds great promise. Nevertheless, the multi-hydroxyl structure of cellulose endows it with strong polarity, leading to poor compatibility with vegetable oils. Accordingly, it requires modification before being used as a thickener for oleogels. The common modification approaches for cellulose include esterification [32], isocyanation [33], and alkylation [34]. For instance, Martín-Alfonso et al. [35] used ethylated cellulose pulp as a thickener to thicken castor oil, and the effect of the degree of ethyl substitution on the rheological properties of the oleogels was investigated. However, traditional modification methods involve the use of numerous organic solvents, which not only increase production costs but also cause serious harm to human health. Therefore, it is urgent to develop a novel, facile, and environmentally friendly approach for preparing cellulose-based oleogels.

The multi-hydroxyl structure of cellulose exhibits high reactivity toward isocyanate groups (-NCO), owing to the strong nucleophilicity of the oxygen atom in the hydroxyl groups. This facilitates efficient nucleophilic addition reactions between the two components. Epoxy soybean oil (ESO), a biodegradable, low-toxicity bio-based plasticizer derived from renewable vegetable oils, contains reactive epoxy groups in its molecular structure that are also capable of undergoing ring-opening addition reactions with -NCO groups. Leveraging the dual reactive characteristics of cellulose and ESO toward isocyanate, we hypothesize that when ESO is selected as the base oil, the rational incorporation of cellulose and isocyanate into the ESO matrix will trigger a synergistic cross-linking reaction: (1) The polyhydric groups of cellulose will react with -NCO to form urea or carbamate bonds. (2) The epoxy groups of ESO will undergo ring-opening cross-linking with -NCO. Such a dual cross-linking network will effectively immobilize the ESO base oil within the three-dimensional polymer framework, ultimately leading to the formation of a stable bio-based oleogel system with tailorable mechanical and rheological properties. Herein, we developed a novel strategy for preparing oleogels, wherein microcrystalline cellulose (MCC) was used as the thickener, epoxidized soybean oil (ESO) as the base oil, and diphenylmethane diisocyanate (MDI) as the cross-linking agent. The hydroxyl groups on MCC and epoxy groups on ESO react with isocyanate groups, respectively, forming a cross-linking network that thickens the base oil. Notably, ESO not only functions as the base oil for the oleogels but also acts as a solvent for the cross-linking reaction, thus eliminating the need for organic solvents. We systematically characterized the physicochemical, rheological, and tribological properties of the cellulose-based oleogels with varying cross-linking densities, which can be modulated by adjusting the MCC/MDI ratio. This work offers novel insights for the development of eco-friendly oleogels.

## 2. Results

### 2.1. Synthesis and Characterization of Oleogels

**Figure 1:**
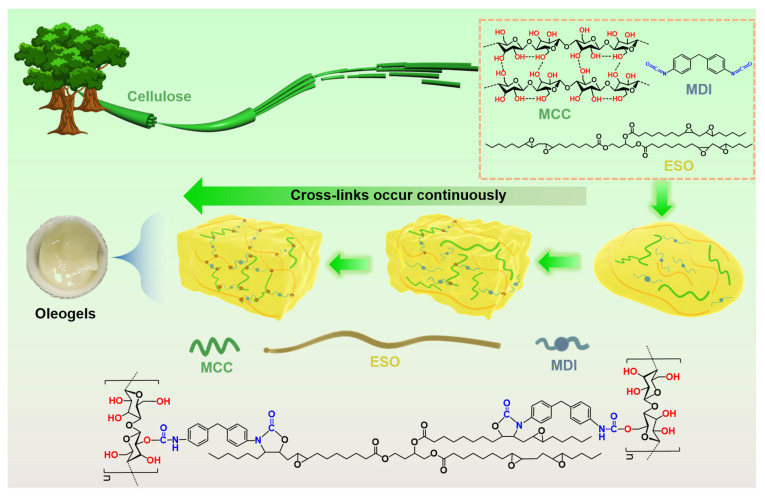
Schematic diagram of the preparation process and cross-linking structure of ESO/MCC-MDI oleogels.

The preparation process of the oleogels is illustrated in Figure 1. Unlike conventional oleogel preparation methods, this work employs epoxidized soybean oil (ESO) as both the reaction medium and base oil, thus avoiding the use of organic solvents and enabling a solvent-free green synthesis route. During the reaction, both the epoxy groups of ESO and the hydroxyl groups (-OH) on the microcrystalline cellulose (MCC) chains can react with the isocyanate groups (-NCO) of diphenylmethane diisocyanate (MDI), forming a stable three-dimensional cross-linked network among the three components. This cross-linking strategy not only resolves the incompatibility arising from the polarity difference between MCC and ESO but also enables precise tuning of the oleogel rheological properties. More importantly, the apparent cross-linking density can be tailored by adjusting the mass ratio of MDI to MCC, enabling systematic control over the mechanical strength, thermal stability, oxidation resistance, and lubrication performance of the oleogels. Notably, the cross-linking reaction continues gradually over 24 days after preparation, forming a dynamically reinforced network that further enhances the structural stability of the oleogels. Compared with conventional greases using metal soap thickeners, the metal-free, renewable, bio-based oleogel system developed in this work shows strong potential for green lubrication applications.

**Figure 2:**
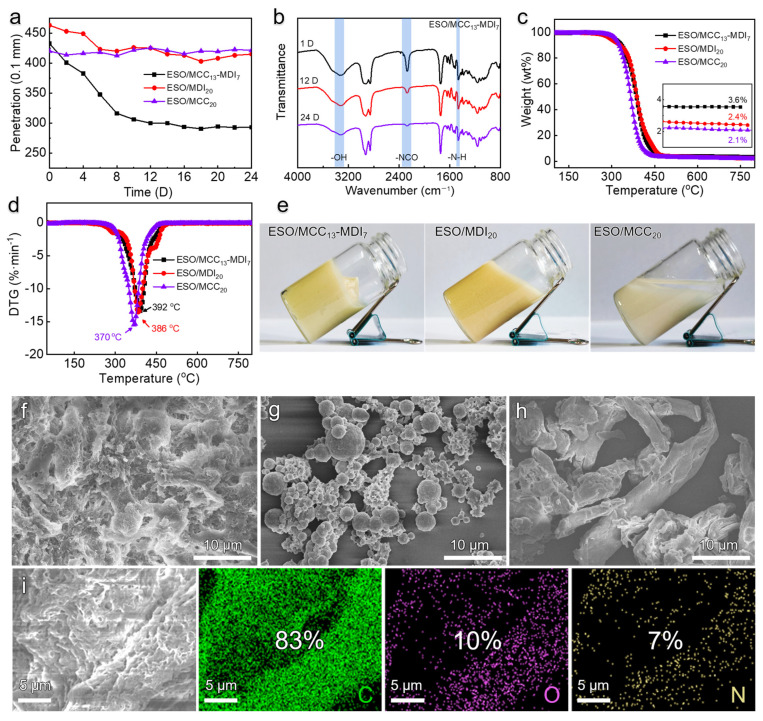
(**a**) Penetration values of ESO/MCC_13_-MDI_7_, ESO/MDI_20_, and ESO/MCC_20_ versus time; (**b**) FTIR spectra of ESO/MCC_13_-MDI_7_ with different cross-linking times; (**c**) TG and (**d**) DTG curves of ESO/MCC_13_-MDI_7_, ESO/MDI_20_, and ESO/MCC_20_; (**e**) digital photographs of ESO/MCC_13_-MDI_7_, ESO/MDI_20_, and ESO/MCC_20_ after 14 days of static storage; (**f**–**h**) SEM images of ESO/MCC_13_-MDI_7_, ESO/-MDI_20_, and ESO/MCC_20_, respectively; (**i**) EDS elemental mapping of the surface of ESO/MCC_13_-MDI_7_.

Figure 2a shows that the penetration value of ESO/MCC_13_-MDI_7_ decreases gradually with storage time, indicating that continuous cross-linking occurs and progressively increases the oleogel hardness. Specifically, the penetration value of ESO/MCC_13_-MDI_7_ declines steadily from 432 (0.1 mm) to 293 (0.1 mm) over 24 days of standing. By comparison, ESO/MDI20 exhibits a slower decrease in penetration value and remains at a higher level, owing to its lower cross-linking density. For ESO/MCC_20_, the penetration value also shows little change during storage, because no covalent cross-links can form between MCC and ESO, which only exist as a physical blend. The FTIR spectra (Figure 2b) show that the absorption peaks assigned to -NCO and -OH groups in ESO/MCC_13_-MDI_7_ decrease with increasing preparation time, while the peak corresponding to -N-H moiety gradually increases. This provides strong evidence that the cross-linking reaction continues to proceed even after the completion of oleogel preparation. Other ESO/MCC-MDI oleogel samples with different ratios of MCC and MDI also exhibit a similar trend in their FTIR absorption peaks (Figure S1). The thermal decomposition of the oleogels was investigated by TGA, and the relevant data are listed in Table S1. These data indirectly reflect the effect of chemical cross-linking on the thermal stability of the oleogels. As shown in Figure 2c, the char yield (*Y*_c_) of ESO/MCC_13_-MDI_7_ increases to 3.6%, compared to 2.4% for ESO/MDI_20_ and 2.1% for ESO/MCC_20_. This increase is attributed to the stronger cross-linked network formed in ESO/MCC_13_-MDI_7_, which more effectively restricts the escape of volatile degradation products during pyrolysis, thereby promoting carbonization and char formation. The DTG curves (Figure 2d) show that the maximum decomposition temperature (*T*_max_) of ESO/MCC_13_-MDI_7_ rises to 392 °C, compared to 386 °C for ESO/MDI_20_ and 370 °C for ESO/MCC_20_. These results demonstrate that the chemical cross-linking structure in ESO/MCC_13_-MDI_7_ effectively improves the thermal stability of the oleogel. Figure 2e shows digital photographs of ESO/MCC_13_-MDI_7_, ESO/MDI_20_, and ESO/MCC_20_ after 14 days of static storage. It can be observed that ESO/MCC_13_-MDI_7_ and ESO/MDI_20_ maintain good phase stability after storage, whereas ESO/MCC_20_ undergoes phase separation due to the large polarity difference between MCC and ESO. These control experiments provide strong indirect evidence that a covalent network formed via simultaneous –OH/NCO and epoxy/NCO reactions is essential for the formation of a stable oleogel. Neither MCC alone nor MDI alone is sufficient.

### 2.2. Physicochemical Properties

**Figure 3:**
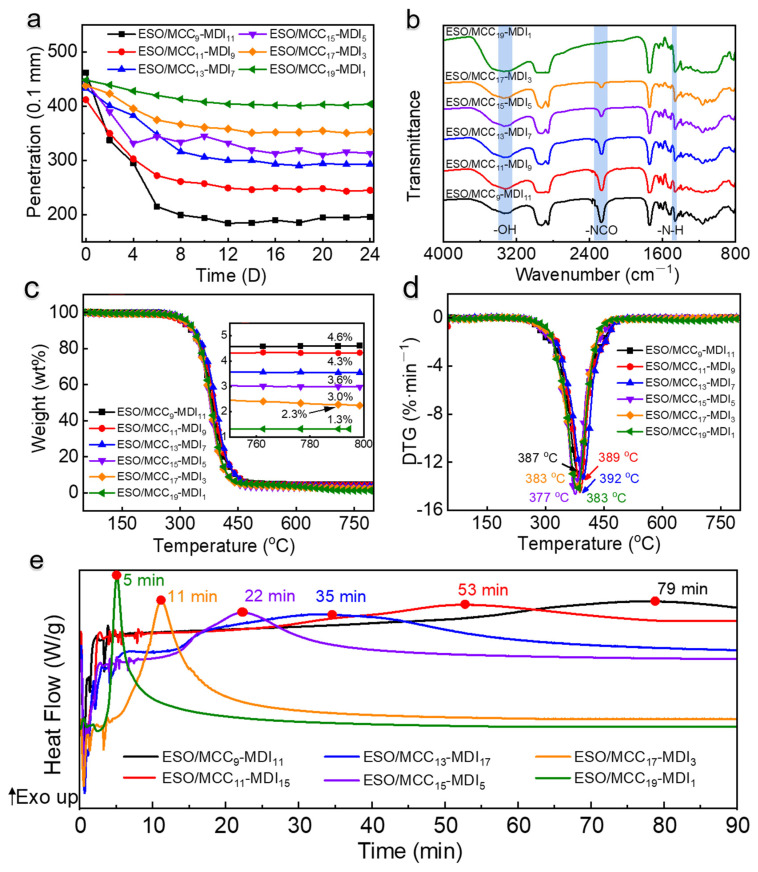
(**a**) Penetration value changes of oleogels with different cross-linking densities versus time; (**b**) FTIR spectra, (**c**) TG and (**d**) DTG curves, and (**e**) OIT curves of oleogels with different cross-linking densities at 210 °C.

The cross-linking reaction among ESO, MDI, and MCC ultimately produces a thickening effect, leading to the formation of oleogels. As a key structural factor, the cross-linking density directly governs the physicochemical properties of the as-prepared oleogels. In this work, five groups of oleogels with varying cross-linking densities were designed by adjusting the mass ratio of MDI to MCC. The penetration test results are presented in Figure 3a. As the standing time increases, the penetration value of all oleogel samples gradually decreases and reaches equilibrium. The minimum penetration values are summarized in Table 1. It can be observed that as the MDI content rises, the penetration value of the oleogels decreases from 404 (0.1) to 196 (0.1 mm), and the NLGI grade climbs from 0 to 4. Therefore, the cross-linking degree of the oleogels can be tailored to obtain the target penetration value via regulating the MDI-to-MCC ratio. Figure 3b presents the FTIR spectra of various oleogels recorded on the first day after preparation, where the intensity of the characteristic peak of -NCO group is observed to rise with increasing MDI content. This phenomenon is attributed to the introduction of more -NCO groups into the oleogel matrix at higher MDI loadings. Figure 3c,d presents the TG curves of the oleogels, with related thermal parameters summarized in Table S1. The results reveal that the *Y*_c_ of the oleogels at 800 °C is elevated with increasing cross-linking density, improving from 1.3% for ESO/MCC_19_-MDI_1_ to 4.6% for ESO/MCC_9_-MDI_11_, confirming that the cross-linking reaction effectively enhances the char-forming ability of the oleogels. The Tmax of all samples are relatively close, because the thermal decomposition of the 80 wt% base oil dominates the main mass loss stage. In contrast, the *Y*_c_ is mainly determined by the cross-linked polymer network and carbonaceous residue from MCC and MDI, thus showing a remarkable increase with elevated cross-linking density. The 5% weight loss temperature (*T*_5%_) is a key indicator for evaluating the initial thermal stability and practical service limit of the oleogels. As listed in Table S1, all samples exhibit a *T*_5%_ around 300 °C, indicating a high upper service temperature limit and excellent thermal stability. The drop point is defined as the temperature at which an oleogel undergoes a phase transition from semisolid to liquid. During drop point measurements, all oleogel samples exceeded the instrument’s upper temperature limit (330 °C), preventing the determination of their exact drop point values. This result unambiguously confirms their excellent high-temperature stability. The oil separation rates of different oleogels are summarized in Table 1. The results illustrate that the oil separation rate declines with increasing cross-linking density. Among these samples, ESO/MCC_19_-MDI_1_ could not be measured due to its excessive fluid consistency. The ESO/MCC_9_-MDI_11_ sample shows the lowest oil separation rate of 0.02%, which is 98.2% lower than the highest value of 1.12% observed for ESO/MCC_17_-MDI_3_.

### 2.3. Antioxidant Properties

The antioxidant effect of oleogels was evaluated by oxidative induction time (OIT) measurements as described in a previous study [25]. Free radicals are generated when materials are exposed to high-temperature oxygen in OIT experiments. Oil oxidation is an exothermic process that releases heat, as shown in Figure 3e. OIT increases with cross-linking density. Specifically, the OIT of ESO/MCC_19_-MDI_1_ under pure oxygen at 210 °C is the shortest (only 5 min), whereas that of ESO/MCC_9_-MDI_11_ reaches the maximum value of 79 min. Furthermore, all oleogels exhibit significantly superior oxidation resistance compared to pure ESO (OIT = 4.5 min), as shown in Figure S2. This enhancement is attributed to the dense three-dimensional covalent network formed by cross-linking, which acts as a physical barrier that hinders the penetration and diffusion of oxygen into the bulk oil phase. As cross-linking density increases, the network becomes more compact, further limiting oxygen availability for initiating or propagating oxidation reactions. The improved antioxidant performance is critical for extending the service life and enhancing the high-temperature stability of the oleogels.

### 2.4. Rheological Behavior

**Figure 4:**
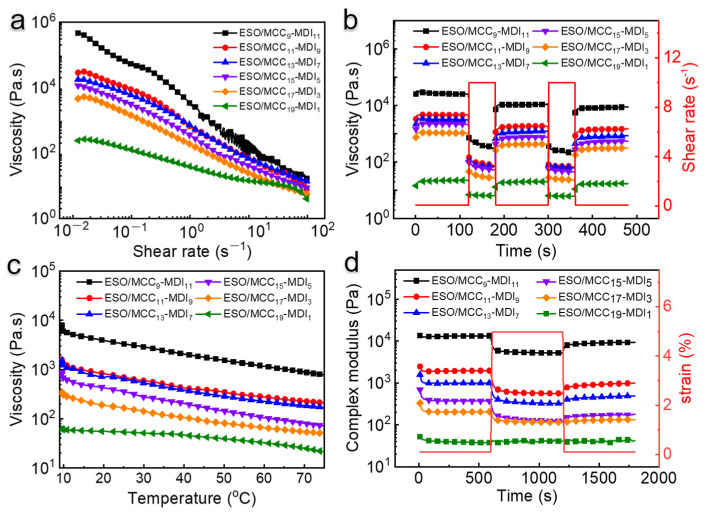
(**a**) Viscosity–shear rate profiles, (**b**) thixotropic properties, (**c**) viscosity–temperature sweep curves, and (**d**) structural recovery behaviors of oleogels with different cross-linking densities.

**Viscosity-shear rate sweep:** Oleogels should possess suitable flowability to satisfy lubrication requirements. As shown in Figure 4a, viscosity-shear rate sweep tests were carried out to evaluate the flow behavior of the oleogels. The viscosity of all oleogels decreases with the increase in the shear rate, reflecting significant shear-thinning behavior. At a given shear rate, the viscosity increases with the cross-linking density of the oleogels. The jitter observed in ESO/MCC_9_-MDI_11_ under high shear is primarily caused by wall slip between the oleogel and the rheometer plates [36].

**Thixotropic properties:** The thixotropic behavior of the oleogels was further investigated by monitoring viscosity changes at low and high shear rates (0.1 and 10 s^−1^, respectively) over a fixed time interval. As illustrated in Figure 4b, the viscosity decreases significantly with increasing shear rate, indicating that the gel network is substantially disrupted. Nevertheless, the structure shows partial recovery when the shear rate is returned to a low level. These results confirm that the oleogels exhibit thixotropic behavior, characterized by distinct shear thinning and partial structural recovery. This unique thixotropic property is favorable for lubrication applications that require reversible structural disruption and recovery [37].

**Viscosity-temperature sweep:** Figure 4c depicts the effect of temperature on the viscosity of oleogels with different cross-linking densities. It can be found that the viscosity of all oleogels decreases with increasing temperature, which is attributed to the intensification of molecular thermal motion that weakens intermolecular interactions and flow resistance. Notably, viscosity is positively correlated with cross-linking density, indicating that cross-linking enhances intermolecular interactions, thereby restricting molecular mobility and increasing flow resistance.

**Structural recovery:** Figure 4d shows the evolution of the complex modulus (G*) for oleogels with different cross-linking densities, which combines contributions from the elastic modulus (*G′*) and viscous modulus (*G″*). The G* decreases when a shear strain outside the linear viscoelastic range (LVR) is applied on the oleogels; subsequently, partial or total recovery in the G* is observed when a shear strain inside the LVR is restored again. The percentage values of recovery and destruction for the oleogels were calculated according to a previous publication [38] as follows:

(1) $$\mathrm{Destruction}\;\mathrm{ratio}\;=\frac{G_{0}^{*}-G_{1}^{*}}{G_{0}^{*}}\times \;100\%$$

(2) $$\mathrm{Recovery}\;\mathrm{ratio}=\frac{G_{2}^{*}-G_{1}^{*}}{G_{0}^{*}-G_{1}^{*}}\times 100\%$$

where $G_{0}^{*}$ is the complex modulus for the first shear strain applied (inside the LVR, strains = 0.1%); $G_{1}^{*}\;$ is the complex modulus for the second shear strain applied (outside the LVR, strains = 5%); and $G_{2}^{*}$ is the complex modulus for the third shear strain value (inside the LVR, strains = 0.1%). The relevant data are listed in Table 2. The destruction and recovery ratios of ESO/MCC_19_-MDI_1_ could not be calculated due to the absence of structural strength. For the other oleogels, both the destruction ratio and recovery ratio decrease with decreasing cross-linking density, because a lower cross-linking density results in a less rigid and compact network. Thus, ESO/MCC_9_-MDI_11_ possesses the highest structural recovery ratio of 44.7%.

**Figure 5:**
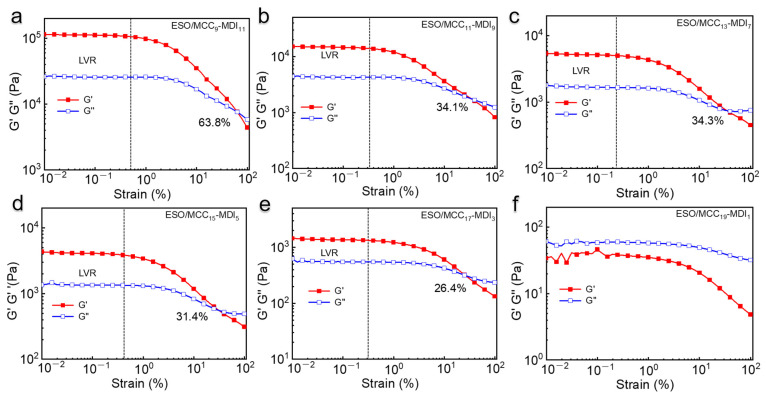
Evolution trends of *G′* and *G″* for oleogels with different cross-linking densities: (**a**) ESO/MCC_9_-MDI_11_, (**b**) ESO/MCC_11_-MDI_9_, (**c**) ESO/MCC_13_-MDI_7_, (**d**) ESO/MCC_15_-MDI_5_, (**e**) ESO/MCC_17_-MDI_3_, and (**f**) ESO/MCC_19_-MDI_1_.

**Viscoelasticity:** Figure 5 presents the viscoelastic properties of oleogels with different cross-linking densities, which are critical for evaluating their gel network stability and application potential as lubricating materials. The storage modulus (*G′*) is an indicator of the elastic nature of oleogels, corresponding to their solid-like behavior and the energy stored during shear deformation. In contrast, the loss modulus (*G″*) characterizes the viscous response of the system, representing the liquid-like behavior and the energy dissipated under shear conditions [39]. As shown in Figure 5f, the viscoelastic behavior of ESO/MCC_19_-MDI_1_ differs significantly from that of other samples: *G″* is consistently higher than *G′* across the entire strain range, indicating a dominant fluid-like behavior. This observation is inconsistent with the typical rheological characteristics of gel materials, which further confirms that no stable oleogel network can be formed when the cross-linking density is excessively low. This phenomenon can be attributed to the insufficient cross-linking points to maintain a stable three-dimensional network structure, leading to the failure of gel formation. For all other oleogel samples (with moderate to high cross-linking densities), a clear linear viscoelastic region (LVR) is identified. The LVR was defined as the strain range over which the storage modulus (*G′*) remained constant within ±5% of its initial value. Within this region, both *G′* and *G″* remain nearly independent of strain, suggesting that the oleogels retain a stable gel network structure under small-strain shear conditions [40]. Beyond the LVR, with the increase in strain, *G′* gradually decreases while *G″* increases, eventually resulting in a cross-over (flow point) between the two moduli. This cross-over point is a key rheological parameter, as it marks the onset of gel network breakdown and the transition from gel-like to liquid-like behavior. Notably, the flow point shifts toward higher strain values with the increase in cross-linking density, indicating that oleogels with higher cross-linking densities possess stronger resistance to shear-induced structural damage. Among all samples, ESO/MCC_9_-MDI_11_ exhibits the most pronounced solid-like character, with a solid-like fraction of 63.8% (Figure 5a–e). This result further verifies that increasing cross-linking density enhances the elastic properties and structural stability of the oleogel network, which is consistent with the fundamental principle that higher cross-linking density promotes the formation of a more compact and robust three-dimensional network.

**Figure 6:**
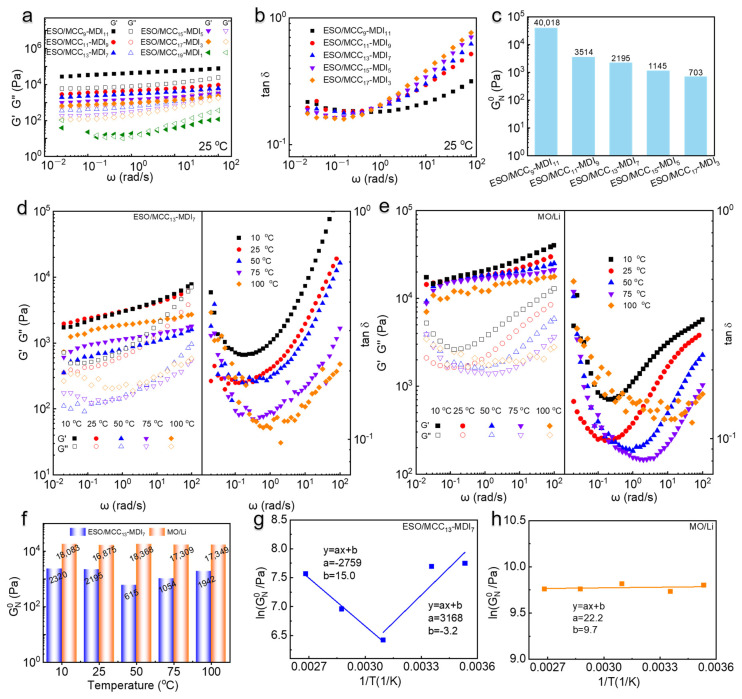
(**a**,**b**) Evolution of *G′*, *G″*, and tan δ versus frequency; (**c**) $G_{N}^{O}$ of oleogels with different cross-linking densities; (**d**,**e**) evolution of *G′*, *G″* and tan δ of ESO/MCC_13_-MDI_7_ and MO/Li versus frequency at different temperatures; (**f**) $G_{N}^{O}$ of ESO/MCC_13_-MDI_7_ and MO/Li at different temperatures; (**g**,**h**) Arrhenius fitting plots for ESO/MCC_13_-MDI_7_ and MO/Li.

In addition, the frequency dependence of small-amplitude oscillatory shear (SAOS) functions within the LVR at 25 °C for oleogels with different cross-linking densities is also investigated. Consistent with the above results, both *G′* and *G″* increase with increasing cross-linking density. It is worth noting that for the ESO/MCC_19_-MDI_1_ oleogel, G″ exceeds *G′* across the entire frequency range, indicating a viscoelastic liquid-like behavior rather than a gel-like structure. The loss tangent (tan δ) is defined as the ratio of *G″* to *G′*, and the corresponding tan δ values of the oleogels are presented in Figure 6b. For all samples, tan δ decreases initially with increasing frequency, reaches a minimum near ω = 100 rad/s, and then increases, indicating a characteristic relaxation transition. At higher frequencies (ω > 100 rad/s), tan δ decreases with increasing cross-linking density, suggesting that oleogels with higher cross-linking density exhibit a more dominant elastic response under high-frequency oscillatory shear. Figure 6c shows the plateau modulus ($G_{N}^{O}$) of oleogels with different cross-linking densities, defined as the *G′* value at the frequency corresponding to the minimum tan δ. $G_{N}^{O}$ increases with increasing cross-linking density, which is attributed to higher cross-linking density giving rise to stronger chain entanglement.

The evaluation of thermo-rheological behavior is crucial for predicting lubrication performance in grease-lubricated contacts, given that significant frictional heating is inevitably generated in these components under actual operational conditions. To address this, a commercial additive-free lithium-based lubricating grease (MO/Li) was employed as a reference, while ESO/MCC_13_-MDI_7_ was selected as the representative sample to investigate its thermo-rheological characteristics. Figure 6d,e illustrates the evolution of SAOS of ESO/MCC_13_-MDI_7_ as a function of temperature (10–100 °C). A key observation is that *G′* is consistently higher than *G″* across the entire tested frequency range, which is a hallmark of gel-like rheological behavior. Specifically, *G′* increases slightly with increasing frequency, whereas *G″* exhibits a distinct minimum value. These rheological responses are consistent with those of MO/Li grease, confirming that ESO/MCC_13_-MDI_7_ possesses typical gel-like rheological properties, which is essential for its application as a lubricating grease. It is worth noting that the storage modulus of ESO/MCC_13_-MDI_7_ does not exhibit a simple monotonic temperature dependence (Figure 6d). Instead, *G′* first decreases from 10 °C to approximately 50 °C and then increases upon further heating. This non-monotonic behavior is mainly attributed to a thermoreversible sol–gel transition of cellulose in ESO, analogous to the temperature-induced hydrophobic association reported for cellulose derivatives. Above 50 °C, heating disrupts hydrogen bonds among cellulose chains, exposing hydrophobic segments that undergo hydrophobic interactions with each other and with the ESO matrix, forming physical cross-linking nodes that reinforce the network structure. Figure 6f presents the statistical analysis of $G_{N}^{O}$ of both samples at different temperatures. Notably, $G_{N}^{O}$ of MO/Li shows only a slight decrease with increasing temperature, indicating that the reference grease has relatively weak thermal sensitivity and stable network structure under the tested temperature range. In contrast, $G_{N}^{O}$ of ESO/MCC_13_-MDI_7_ exhibits significant thermal responsiveness, decreasing initially and then increasing with the rise in temperature. This unique temperature-dependent behavior suggests that the network structure of ESO/MCC_13_-MDI_7_ is more susceptible to temperature changes compared to MO/Li. To quantitatively evaluate the temperature dependence of $G_{N}^{O}$, an Arrhenius-type equation (Equation (3)) is adopted, as previously reported in the literature [41,42,43]:

(3) $$G_{N}^{O}={Ae}^{E_{a}/RT}$$

where *E*_a_ is the activation energy describing the thermal dependence (J mol^−1^), R is the gas constant (8.314 J mol^−1^ K^−1^), T is the absolute temperature (K), and A is the pre-exponential factor (Pa).

The fitting curves of ESO/MCC_13_-MDI_7_ and MO/Li based on this equation are shown in Figure 6g,h, and the corresponding *E*_a_ values fitted in both low- and high-temperature ranges are summarized in Table 3. The fitting results reveal distinct thermal response behaviors of ESO/MCC_13_-MDI_7_ in the low- and high-temperature ranges. At low temperatures, the *E*_a_ of ESO/MCC_13_-MDI_7_ is 381 kJ mol^−1^, which is much higher than that of MO/Li (22 kJ mol^−1^). This indicates that the network structure of ESO/MCC_13_-MDI_7_ is more sensitive to temperature changes at low temperatures compared to the reference grease. However, when the temperature exceeds 50 °C, the *E*_a_ value of ESO/MCC_13_-MDI_7_ shifts to −331 kJ mol^−1^, while the *E*_a_ of MO/Li remains unchanged. This abnormal negative *E*_a_ value was related to the sol–gel transition of cellulose in ESO. The increase in *G′* and *G″* above 50 °C is attributed primarily to the heating-induced hydrophobic association of cellulose chains, analogous to the thermoreversible sol–gel transition reported for methylcellulose, hydroxypropyl methylcellulose, and ethyl cellulose systems in both aqueous and oil media [44]. At elevated temperatures, the disruption of hydrogen bonds among cellulose chains exposes hydrophobic segments, which then undergo hydrophobic interactions with each other and with the epoxidized soybean oil (ESO) matrix, forming physical cross-linking nodes that reinforce the network structure. The enhanced cross-linking reaction strengthens the network structure of the oleogel, leading to an increase in $G_{N}^{O}$ with increasing temperature.

In summary, the thermo-rheological behavior of ESO/MCC_13_-MDI_7_ demonstrates gel-like characteristics similar to commercial lithium-based grease as well as unique thermal responsiveness. The significant temperature dependence of its $G_{N}^{O}$, especially the abnormal variation at high temperatures, is closely associated with the temperature-induced cross-linking reactions. These findings provide valuable insights into the application potential of ESO/MCC_13_-MDI_7_ as a lubricating grease, particularly under conditions involving temperature fluctuations.

### 2.5. Tribological Properties

**Figure 7:**
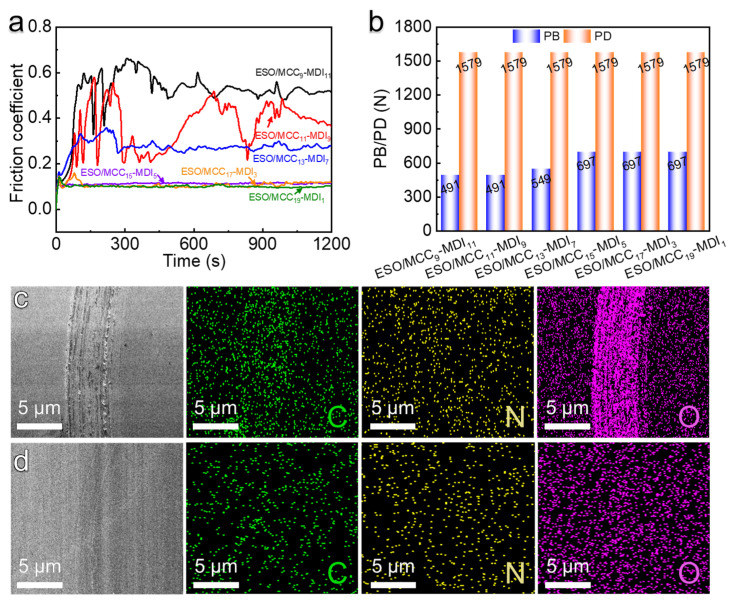
(**a**) Friction coefficients and (**b**) PB and PD values of oleogels with different cross-linking densities; (**c**,**d**) SEM images and EDS elemental mapping of worn surfaces for ESO/MCC_9_-MDI_11_ and ESO/MCC_19_-MDI_1_.

The tribological properties of the oleogels were evaluated using a ball-on-disk tribometer. As illustrated in Figure 7a, the friction coefficient of the oleogels is found to increase with rising cross-linking density. Specifically, the ESO/MCC_19_-MDI_1_ sample exhibits the lowest friction coefficient of 0.100, representing an 80.6% reduction compared to the highest value of 0.515 observed for ESO/MCC_9_-MDI_11_. This phenomenon can be attributed to the fact that oleogels with lower cross-linking densities are easier to release base oil, thereby enabling the formation of lubricating film with lower friction coefficient. Correspondingly, the worn surface morphologies of two representative samples of ESO/MCC_9_-MDI_11_ and ESO/MCC_19_-MDI_1_ were characterized by SEM, as shown in Figure 7c,d. In contrast to ESO/MCC_19_-MDI_1_, the worn surface of ESO/MCC_9_-MDI_11_ appears significantly rougher, featuring deep furrows and evident adhesive wear, indicating severe tribological damage. EDS mapping reveals a high concentration of oxygen elements on the worn surface of ESO/MCC_9_-MDI_11_, suggesting that a severe oxidation reaction occurred between the material and the metal counterpart during the friction process. The extreme pressure (EP) properties of the oleogels were assessed using a four-ball test [45], and the results for the last non-seizure load (PB) and weld point (PD) are presented in Figure 7b. The PB decreases with increasing cross-linking density. This is because a denser network restricts the release of free ESO, resulting in a weaker lubricating film with lower load-bearing capacity. In contrast, the PD remains constant across all samples, as it is determined solely by the intrinsic extreme-pressure properties of the base oil (ESO), which is identical for all oleogels.

Taken together, the ESO/MCC_13_-MDI_7_ sample with a moderate cross-linking density exhibits excellent comprehensive performance (friction coefficient, extreme-pressure performance, rheological properties, thermal stability, and oil retention), rendering it a promising candidate for practical lubrication applications.

## 3. Materials and Methods

### 3.1. Materials

Epoxidized soybean oil (≥99.5%) was purchased from Shanghai Aladdin Biochemical Technology Co., Ltd. (Shanghai, China), Microcrystalline cellulose (particle diameter: 25 μm) and diphenylmethane diisocyanate (≥98%) were purchased from Shanghai Maclin Biochemical Technology Co., Ltd. (Shanghai, China).

### 3.2. Preparation of Oleogels

Firstly, MDI was dissolved in ESO at 80 °C and stirred with a mechanical stirrer at 150 rpm for 5 min. Next, MCC was slowly added to the ESO solution. Then, the mixture was heated to 150 °C and stirred for another 30 min. Finally, the resulting mixture was cooled to room temperature and ground 3–5 times using a three-roll mill to obtain the cellulose-based oleogels (ESO/MCC_x_-MDI_y_), where x and y represent the added masses of MCC and MDI during the oleogel preparation, respectively. The detailed compositions of different oleogels are listed in Table 4 to investigate the effects of different chemical cross-linking ratios on the properties of the oleogels.

### 3.3. Characterization of Oleogels

Microstructural characterization of oleogels was performed using a scanning electron microscope (SEM, Hitachi SU8010, Tokyo, Japan). Prior to analysis, an appropriate amount of oleogel sample was placed onto a flat monocrystalline silicon wafer and immersed in petroleum ether for 24 h to remove the oil phase. Subsequently, the sample was sputter-coated with gold. The chemical structures of oleogels were characterized by Fourier transform infrared spectroscopy (FTIR, Brucker Vertex-70, Bremen, Germany). The thermal stability of the oleogels was evaluated using a Q-500 thermogravimetric analyzer (NETZSCH Instruments, Bavaria, Germany). The samples were heated from 30 to 800 °C at a heating rate of 10 °C/min under a nitrogen atmosphere. The oxidation resistance of the oleogels was assessed by determining the oxidation induction time (OIT). The OIT was determined using a differential scanning calorimeter (DSC) (FPB-O, NETZSCH Instruments) according to the SH/T 0790-2007 standard [46]. Samples were heated from room temperature to 210 °C under an oxygen atmosphere. The time corresponding to the onset of the oxidation exothermic peak was recorded as the OIT.

### 3.4. Physicochemical Properties Test

The dropping point of the oleogels was determined using a drop point apparatus (WQD-1A, Shanghai INESA Physico Optical Instrument Co., Ltd., Shanghai, China) in accordance with the ASTM D566 standard [47]. The temperature at which the first droplet fell was recorded. The consistency grade was assessed using a penetrometer (SYD-2801 C, Shanghai Changji Geological Instrument Co., Ltd., Shanghai, China) according to the GB/T 269-2023 standard [48]. The oil separation rate was measured following the NB/SH/T 0324-2010 standard [49]. Specifically, oleogel samples were heated in an oven at 100 °C for 24 h, and the proportion of base oil lost was calculated as the oil separation rate.

### 3.5. Rheological Behaviors

The rheological behavior of the oleogels was investigated using a controlled-stress rheometer (ARES-G2, TA Instruments, New Castle, DE, USA). All measurements were performed using a plate–plate geometry (25 mm diameter, 1 mm gap). Shear rate sweep tests were conducted to measure the viscosity under steady shear flow over a shear rate range of 0.01–100 s^−1^ at 25 °C. Temperature ramp tests were performed to investigate the effect of temperature on viscosity. Measurements were conducted in rotational mode at a constant shear rate of 1 s^−1^ over a temperature range of 10–80 °C. Thixotropic behavior was evaluated by monitoring the time-dependent viscosity under alternating low (0.1 s^−1^) and high (10 s^−1^) shear rates. The initial transient effects (within the first few seconds) were excluded from data analysis to avoid instrument inertia and sample loading artifacts. The stable viscosity values at each step were taken as the meaningful rheological responses. Structural recovery was investigated using a three-step strain protocol. The sample was first subjected to a strain (0.1%) within the linear viscoelastic region (LVR), followed by a high destructive strain of 5%, and finally returned to the initial LVR strain to monitor the recovery of the viscoelastic properties. Oscillatory shear measurements were conducted to obtain the storage modulus (*G′*), loss modulus (*G″*), and loss tangent (tan δ). Amplitude sweeps were performed from 0.01% to 100% shear strain at a constant angular frequency of 10 rad/s to determine the linear viscoelastic region (LVR, within ±5% of its initial value) and the flow point (cross-over between *G′* and *G″*). Small-amplitude oscillatory shear (SAOS) tests were performed within the linear viscoelastic region at frequencies from 0.01 to 100 rad/s under various fixed temperatures (10, 25, 50, 75, 100 °C). All tests were performed after the oleogels had reached a stable state. All rheological measurements were performed in triplicate on independently prepared samples. The representative curves shown in the figures were selected after confirming good reproducibility.

### 3.6. Tribology Tests

The friction coefficients of the oleogels were measured using a UMT-2 tribometer (Bruker, Billerica, MA, USA) equipped with a ball-on-disk configuration. The steel ball (GCr15 steel, 6 mm diameter) slid on a steel disk lubricated with a 1 mm thick oleogel layer at a linear speed of 15 mm/s under a load of 30 N for 20 min at 25 °C. The extreme pressure properties were evaluated using a four-ball tester (Hengxu SGW-10A, Jinan HengXu Testing Machine Technology Co., Ltd., Jinan, Shandong, China) in accordance with the GB/T 3142-2019 standard [50]. Each test involves a rotating upper steel ball sliding against three stationary lower balls clamped together in a pot. The pot was filled with sufficient oleogel to fully cover the contact points. For each sample, the upper ball was rotated at 1450 ± 50 r/min at 25 °C for 10 s under a gradually increased load until welding occurred. Triplicate measurements were performed at each load to determine the last non-seizure load (PB) and the weld point (PD). The PB is defined as the highest applied load at which no seizure occurs, while the PD is the lowest load at which welding takes place. All tests were performed after the oleogels had reached a stable state.

## 4. Conclusions

In this work, cellulose-based oleogels with tunable rheological properties were developed via a one-step MCC–MDI–ESO cross-linking strategy. The main findings are summarized as follows:

(1) Cross-linking density controls material properties and enhances stability. As the nominal cross-linker content increases, unworked penetration decreases from 404 to 196 (0.1 mm), oil separation drops to 0.02%, and char yield improves from 1.3% to 4.6%. All oleogels exhibit initial decomposition temperatures around 300 °C, and OIT increases substantially from 5 to 79 min at 210 °C with increasing cross-linking density.
(2) Rheological behavior shows trade-offs. The oleogels exhibit shear-thinning and thixotropic behavior, with plateau modulus and structural recovery improving with cross-linking density. However, recovery ratios are moderate (up to 44.7%).
(3) Tribological performance presents a limitation. Friction coefficient increases with cross-linking density (from 0.100 to 0.515), as denser networks restrict oil release. PB decreases with cross-linking density, while PD remains constant.
(4) Optimal candidate. Considering all properties, ESO/MCC_13_-MDI_7_ (NLGI 2, oil separation 0.09%, OIT 38 min) offers the best overall balance for lubrication applications.

## Figures and Tables

**Table 1:** The physicochemical properties of oleogels with different cross-linking densities.

Sample	Penetration Value (0.1 mm)	NLGI Grade	Oil Separation Rate (%)
ESO/MCC_9_-MDI_11_	196 ± 1	4	0.02
ESO/MCC_11_-MDI_9_	245 ± 2	3	0.04
ESO/MCC_13_-MDI_7_	293 ± 1	2	0.09
ESO/MCC_15_-MDI_5_	313 ± 3	1	0.15
ESO/MCC_17_-MDI_3_	353 ± 1	0	1.12
ESO/MCC_19_-MDI_1_	404 ± 3	0	/

**Table 2:** Viscoelasticity-related parameters of oleogels with different cross-linking densities.

Sample	Destruction Ratio (%)	Recovery Ratio (%)
ESO/MCC_9_-MDI_11_	58.4	44.7
ESO/MCC_11_-MDI_9_	68.8	22.5
ESO/MCC_13_-MDI_7_	65.0	16.2
ESO/MCC_15_-MDI_5_	64.0	11.8
ESO/MCC_17_-MDI_3_	44.5	9.2
ESO/MCC_19_-MDI_1_	/	/

**Table 3:** The activation energy values of ESO/MCC_13_-MDI_7_ and MO/Li.

Sample	*E*_a_ (kJ mol^−1^)	
ESO/MCC_13_-MDI_7_	10–50 °C	50–100 °C
	381	−331
MO/Li	22	

**Table 4:** Compositions of the prepared oleogels.

Sample	ESO (g)	MCC (g)	MDI (g)
ESO/MDI_20_	80	0	20
ESO/MCC_20_	80	20	0
ESO/MCC_9_-MDI_11_	80	9	11
ESO/MCC_11_-MDI_9_	80	11	9
ESO/MCC_13_-MDI_7_	80	13	7
ESO/MCC_15_-MDI_5_	80	15	5
ESO/MCC_17_-MDI_3_	80	17	3
ESO/MCC_19_-MDI_1_	80	19	1

## Data Availability

The data presented in this study are available upon request from the corresponding author.

## Supplementary Materials

The following supporting information can be downloaded at: https://www.mdpi.com/article/10.3390/molecules31142538/s1, Figure S1: FTIR spectra of oleogels with different cross-linking densities at different time; Figure S2: OIT of ESO at 210 °C; Table S1: TG data of the oleogels.

## References

[1] Sun F., Zhang Y., Zhang B., Qiao D., Xie F. (2025). Fibrous protein gels: Nanoscale features governing gelation behavior and gel properties. Adv. Colloid Interface Sci..

[2] Blebea N.-M., Pușcașu C., Vlad R.-A., Hancu G. (2025). Chitosan-based gel development: Extraction, gelation mechanisms, and biomedical applications. Gels.

[3] Kothuri V., Han J.H., Keum D.H., Kwon H.C., Kim D.H., Han S.G. (2025). Utilization of emulsion gels in plant-based meat analog formulations: A review. Food Hydrocoll..

[4] Günal-Köroğlu D., Gultekin Subasi B., Saricaoglu B., Karabulut G., Capanoglu E. (2024). Exploring the frontier of bioactive oleogels in recent research. Trends Food. Sci. Technol..

[5] Zampouni K., Sideris N., Tsavdaris E., Katsanidis E. (2024). On the structural and mechanical properties of mixed coconut and olive oil oleogels and bigels. Int. J. Biol. Macromol..

[6] Wang K., Zhang J., Fu Z., Luo Y., Pu C., Tang W., Sun Q. (2024). Oleogels based on peanut protein isolate fibrils: Structural characterization dependent on induction time and suitability in marguerite biscuits. Food Hydrocoll..

[7] Wang T., Wang Y., Wang X., He B., Liu S., Ye Q., Zhou F., Liu W. (2024). Fabrication of ionic supramolecular oleogel lubricants enhanced with liquid metal nanodroplets for superior tribological performance. ACS Nano.

[8] Yu B., Shen Y., Li L., Fan J., Qu J., Xu J. (2026). Ultra-low friction with SiO_2_ nanoparticles bonded sorbitan monostearate oleogel as green lubricant additives. Tribol. Int..

[9] Sathwik Chatra K.R., Osara J.A., Lugt P.M. (2022). Impact of grease churning on grease leakage, oil bleeding and grease rheology. Tribol. Int..

[10] Lugt P.M. (2016). Modern advancements in lubricating grease technology. Tribol. Int..

[11] Wallace T., Gibbons D., O’Dwyer M., Curran T.P. (2017). International evolution of fat, oil and grease (FOG) waste management—A review. J. Environ. Manag..

[12] Nassef B.G., Moradi A., Bayer G., Pape F., Abouelkasem Z.A., Rummel F., Schmölzer S., Poll G., Marian M. (2025). Biogenic palm oil-based greases with glycerol monostearate and soy wax: A rheological and tribological study. Results Eng..

[13] Ye J., Zhang J., Li C., Xu H., Sun M., Xu L., Yu L. (2025). Synthesis and lubricating properties of bio-based lubricants from palm oil. ChemPlusChem.

[14] Wu Z., Prakash B., Shi Y. (2025). Synthesizing lignin-based gelators to prepare oleogels used as green and fossil-free greases. Int. J. Biol. Macromol..

[15] Boruah U., Mohan B., Choudhury N.D., Chowdhury D. (2025). Surface-modified graphitic carbon nitride as a lubricant additive in bio-based oil. ACS Appl. Nano Mater..

[16] Liu Z., Biresaw G., Biswas A., Cheng H.N. (2018). Effect of polysoap on the physical and tribological properties of soybean oil-based grease. J. Am. Oil. Chem. Soc..

[17] Fang Y., Lou G., Wu Q., Cheng X., Chen Y. (2025). Bio-based grease from agricultural waste: Modified cellulose from corn stover for sustainable lubrication. Materials.

[18] Ursachi C.-Ș., Perța-Crișan S., Tolan I., Chambre D.R., Chereji B.-D., Condrat D., Munteanu F.-D. (2024). Development and characterization of ethylcellulose oleogels based on pumpkin seed oil and rapeseed oil. Gels.

[19] Adhvaryu A., Erhan S.Z., Perez J.M. (2004). Preparation of soybean oil-based greases: Effect of composition and structure on physical properties. J. Agric. Food. Chem..

[20] Rawat S.S., Harsha A.P., Khatri O.P. (2022). Tribological investigations of two-dimensional nanostructured lamellar materials as additives to castor-oil-derived lithium grease. J. Tribol..

[21] Xia T., Huang Y., Lan P., Lan L., Lin N. (2019). Physical modification of cellulose nanocrystals with a synthesized triblock copolymer and rheological thickening in silicone oil/grease. Biomacromolecules.

[22] Delgado M., Cortés-Triviño E., Valencia C., Franco J. (2020). Tribological study of epoxide-functionalized alkali lignin-based gel-like biogreases. Tribol. Int..

[23] Sánchez R., Stringari G., Franco J., Valencia C., Gallegos C. (2011). Use of chitin, chitosan and acylated derivatives as thickener agents of vegetable oils for bio-lubricant applications. Carbohydr. Polym..

[24] Gallego R., Arteaga J., Valencia C., Franco J. (2013). Chemical modification of methyl cellulose with HMDI to modulate the thickening properties in castor oil. Cellulose.

[25] Wu Z., Thoresen P.P., Matsakas L., Rova U., Christakopoulos P., Shi Y. (2023). Facile synthesis of lignin-castor oil-based oleogels as green lubricating greases with excellent lubricating and antioxidation properties. ACS Sustain. Chem. Eng..

[26] Yamaji A., Okuda Y., Kobayashi C., Kurahashi R., Kazuma K., Chiba K., Hirata M., Ikemoto Y., Osaka K., Gao J. (2025). Structural analysis of regenerated cellulose textile covered with cellulose nano fibers. Polymers.

[27] Silva F.A., Branco S., Dourado F., Neto B., Gama M. (2025). Life cycle assessment of bacterial cellulose and comparison to other cellulosic sources. J. Clean. Prod..

[28] Simon J., Fliri L., Schlapp-Hackl I., Rosenau T., Potthast A., Hummel M. (2025). Influence of dialcohol cellulose moieties on the properties of cellulosic fibers prepared by the Ioncell^®^ process. Cellulose.

[29] Siripongpreda T., Noikorn N., Phookum T., Suea-Ngam A., Brack E., Ummartyotin S., Rodthongkum N. (2025). Cellulose nanofibrils and semi-interpenetrating recycled cellulose/carboxymethyl cellulose hydrogel integrated with 3D-printed device as a multiplex sensing of pesticides and pH of water. Int. J. Biol. Macromol..

[30] Yekta R., Abedi-Firoozjah R., Azimi Salim S., Khezerlou A., Abdolmaleki K. (2023). Application of cellulose and cellulose derivatives in smart/intelligent bio-based food packaging. Cellulose.

[31] Cheng H., Lijie L., Wang B., Feng X., Mao Z., Vancso G.J., Sui X. (2020). Multifaceted applications of cellulosic porous materials in environment, energy, and health. Prog. Polym. Sci..

[32] Xing W., Wang Z., Zhang K., Xu Y., Pan Y., Zhang G. (2025). Flame-retardant and antibacterial multifunctional cellulose fibers with carbamate esterification and phosphorylation modification. Ind. Crops Prod..

[33] Aziz T., Farid A., Haq F., Kiran M., Ullah A., Zhang K., Li C., Ghazanfar S., Sun H., Ullah R. (2022). A review on the modification of cellulose and its applications. Polymers.

[34] Zheng C., Du X., Wang Q., Yan J., Zhang Y., Zhang X., Wang Y., Wang Z., Zhang L. (2025). Improving thickening and emulsification performances of cellulose nanocrystals by alkylation modification for enhanced oil recovery. Int. J. Biol. Macromol..

[35] Martín-Alfonso J., Núñez N., Valencia C., Franco J., Díaz M. (2011). Formulation of new biodegradable lubricating greases using ethylated cellulose pulp as thickener agent. J. Ind. Eng. Chem..

[36] Ren G., Zhou C., Fan X., Zheng M., Wang S. (2022). Investigating the rheological and tribological properties of polyurea grease via regulating ureido amount. Tribol. Int..

[37] Tian C., Xu H., Dong J. (2024). Development of environmentally friendly lubricant using natural resources: Layered double hydroxide gels. Ind. Crops Prod..

[38] Martín-Alfonso J.E., Martín-Alfonso M.J., Valencia C., Cuberes M.T. (2021). Rheological and tribological approaches as a tool for the development of sustainable lubricating greases based on nano-montmorillonite and castor oil. Friction.

[39] Hyun K., Wilhelm M., Klein C.O., Cho K.S., Nam J.G., Ahn K.H., Lee S.J., Ewoldt R.H., McKinley G.H. (2011). A review of nonlinear oscillatory shear tests: Analysis and application of large amplitude oscillatory shear (LAOS). Prog. Polym. Sci..

[40] Saxena A., Kumar D., Tandon N. (2021). Development of eco-friendly nano-greases based on vegetable oil: An exploration of the character via structure. Ind. Crops Prod..

[41] Gallego R., Arteaga J., Valencia C., Díaz M., Franco J. (2015). Gel-like dispersions of HMDI-cross-linked lignocellulosic materials in castor oil: Toward completely renewable lubricating grease formulations. ACS Sustain. Chem. Eng..

[42] Núñez N., Martín-Alfonso J.E., Eugenio M.E., Valencia C., Díaz M.J., Franco J.M. (2012). Influence of eucalyptus globulus kraft pulping severity on the rheological properties of gel-like cellulose pulp dispersions in castor oil. Ind. Eng. Chem. Res..

[43] Borrero-López A.M., Blánquez A., Valencia C., Hernández M., Arias M.E., Franco J.M. (2019). Influence of solid-state fermentation with Streptomyces on the ability of wheat and barley straws to thicken castor oil for lubricating purposes. Ind. Crops Prod..

[44] Sánchez R., Franco J., Delgado M., Valencia C., Gallegos C. (2011). Rheological and mechanical properties of oleogels based on castor oil and cellulosic derivatives potentially applicable as bio-lubricating greases: Influence of cellulosic derivatives concentration ratio. J. Ind. Eng. Chem..

[45] Saxena A., Kumar D., Tandon N., Kaur T., Singh N. (2022). Development of vegetable oil-based greases for extreme pressure applications: An integration of non-toxic, eco-friendly ingredients for enhanced performance. Tribol. Lett..

[46] (2007). Standard Test Method for Oxidation Induction Time of Lubricating Greases by Pressure Differential Scanning Calorimetry.

[47] (2009). Standard Test Method for Dropping Point of Lubricating Grease.

[48] (2023). Determination of Cone Penetration of Lubricating Greases and Petrolatum.

[49] (2010). Standard Test Method for Oil Separation from Lubricating grease.

[50] (2019). Standard test method for determination of load-carrying capacity of lubricants—Four-ball method.

