# Supplementary material for: Cellulose-Based Oleogels via One-Step Cross-Linking for Lubrication
Source: Molecules. 2026 Jul 21;31(14):2538. doi: 10.3390/molecules31142538 (PMC13414524; doi:10.3390/molecules31142538)
Supplement: Supplementary file 1 [file molecules-31-02538-s001.zip › molecules-4402957-supplementary.pdf]

Supporting Information

Cellulose-Based Oleogels via One-Step Cross-Linking for Lubrication

Yuhao Fang[1, 2], Gaobo Lou[1, 2,*], Hongjiang Yu[1, 2], Lina Liu[1, 2,*], Yifan Chen[1, 2,*]

[1] College of Chemistry and Materials Engineering, Zhejiang A&F University, Hangzhou 311300, China; 18839969651@163.com (Y.F.); 13151320460@163.com (H.Y.)

[2] Zhejiang Key Laboratory of Green and Low-Carbon Utilization Technology of Agriculture and Forestry Biomass, Hangzhou 311300, China

* Correspondence: lougaobo@zafu.edu.cn (G.L.); liulina198310@126.com (L.L.); yfchen@zafu.edu.cn (Y.C.)

.

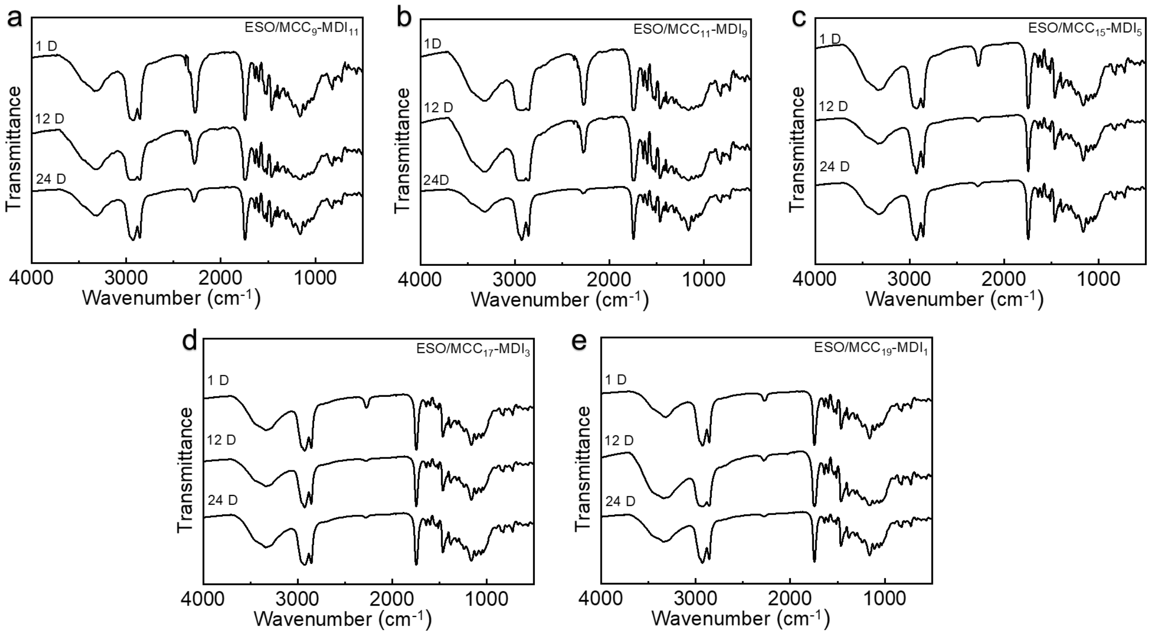

Fig. S1. FTIR spectra of oleogels with different cross-linking densities at different time.

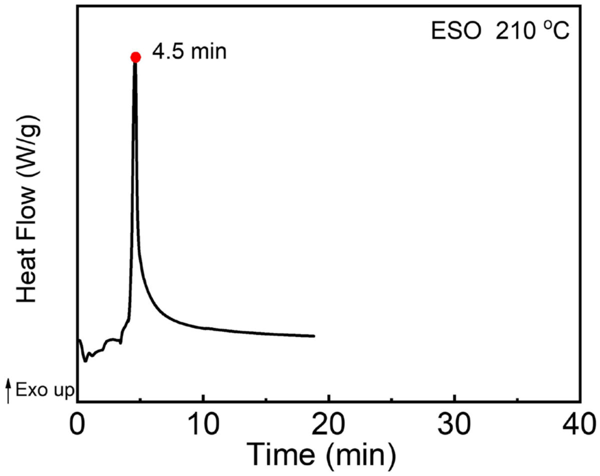

Fig. S2. OIT of ESO at 210 °C

Table S1. TG data of the oleogels.

**Sample**	$T_{5\%}$ (ºC)	T_{max} (ºC)	Y_c (%)
ESO/MDI_{20}	290	386	2.4
ESO/MCC_{20}	307	370	2.1
ESO/MCC_9-MDI_{11}	302	387	4.6
ESO/MCC_{11}-MDI_9	310	389	4.3
ESO/MCC_{13}-MDI_7	299	392	3.6
ESO/MCC_{15}-MDI_5	305	377	3.0
ESO/MCC_{17}-MDI_3	306	383	2.3
ESO/MCC_{19}-MDI_1	317	383	1.3
